# Scoping review protocol of multicomponent interventions to address cardiometabolic disease risk among Pacific Islander children

**DOI:** 10.1371/journal.pone.0280888

**Published:** 2023-01-23

**Authors:** Courtney C. Choy, Kate Nyhan, Kima Savusa, Christina Soti-Ulberg, Rochelle K. Rosen, Take Naseri, Nicola L. Hawley, Mona Sharifi

**Affiliations:** 1 Department of Chronic Disease Epidemiology, Yale School of Public Health, New Haven, Connecticut, United States of America; 2 Harvey Cushing/John Hay Whitney Medical Library, Yale University, New Haven, Connecticut, United States of America; 3 Department of Environmental Health Sciences, Yale School of Public Health, Yale University, New Haven, Connecticut, United States of America; 4 Samoan Obesity, Lifestyle, and Genetic Adaptations Study (OLaGA) Group, Yale School of Public Health, New Haven, Connecticut, United States of America; 5 Ministry of Health, Apia, Samoa; 6 Department of Behavioral and Social Sciences, Brown University School of Public Health, Providence, Rhode Island, United States of America; 7 Center for Behavioral and Preventive Medicine, The Miriam Hospital, Providence, Rhode Island, United States of America; 8 Department of Epidemiology, International Health Institute, Brown University School of Public Health, Providence, Rhode Island, United States of America; 9 Department of Pediatrics, School of Medicine, Yale University, New Haven, Connecticut, United States of America; 10 Department of Biostatistics (Health Informatics), Yale School of Public Health, New Haven, Connecticut, United States of America; Università degli Studi di Milano, ITALY

## Abstract

**Introduction:**

Multicomponent interventions can reduce cardiometabolic disease (CMD) risk factors in childhood; however, little synthesis of the literature has taken place in the Pacific region. Pacific Islanders experience a disproportionately high prevalence of CMD risk factors, yet interventions have been slow to reach many communities. We present this protocol for a scoping review to identify and summarize existing multicomponent interventions to address CMD risk in Pacific Islander children.

**Materials and methods:**

Eligible interventions will (1) address CMD risk factors (including but not limited to obesity, hyperglycemia, dyslipidemia, elevated blood pressure, and/or health behaviors) in 2-to-12-year-old Pacific Islander children, and (2) be multi-component (including at least two lifestyle/behavior change strategies to address CMD risk factors). To investigate existing interventions for adaptation and potential use in Pacific Islander communities, we will search Scopus, MEDLINE ALL (Ovid), EMBASE (Ovid), Yale-licensed Web of Science Core Collection, Cochrane Library, CINAHL (EBSCOhost), ProQuest Dissertations & Theses Global, Global Health (EBSCO), non-indexed Pacific journals, grey literature, government reports, and clinical trial registrations. The Joanna Briggs Institute Manual for Evidence Synthesis and the Preferred Reporting Items for Scoping Reviews will guide data extraction, evidence mapping, synthesis, and reporting of information including study population, intervention components, behavioral changes, health and implementation outcomes, theoretical frameworks, and evaluation measures.

**Ethics and dissemination:**

Formal ethical approval is not required. The dissemination strategy will include peer-reviewed journal publications and presentations. Synthesis of existing multicomponent interventions for Pacific Islander children will help to identify best practices that could be replicated, adapted, or combined.

## Introduction

Multicomponent interventions that use a combination of evidence-based behavioral, psychosocial, and/or environmental change strategies have been developed and implemented to prevent and treat childhood obesity in different population settings [1, 2]. Several recent systematic reviews have reported that effective multicomponent interventions include nutrition (with family involvement and nutrition counseling) and physical activity to improve weight, body mass index (BMI) z-scores, and blood pressure among children with overweight/obesity [2–4]. While many of these interventions have shown efficacy in preventing and treating obesity in the US and other high-come countries [1, 4, 5], there is little evaluation of their implementation in minority and low-income settings [6–8].

Pacific Islanders have among the highest risk of obesity and related cardiometabolic diseases (CMD), yet few interventions and innovations to address CMD risk factors have reached Pacific communities [6, 7, 9–12]. Prevalence of obesity among Pacific Islander children and adolescents aged 2–18 years ranges from 14.7% in the US (National Survey of Children’s Health) to >30% in independent Pacific Island nations [13, 14], which is considerably higher than the US and global averages [14, 15]. Targeting a combination of behavioral and clinical risk factors, including diabetes, dyslipidemia, and/or hypertension, during childhood, interventions developed in other settings will likely need to be adapted or optimized for Pacific Islanders and the proposed context in which they would be delivered. The collectivist structure and practices of Pacific Islander communities (with high levels of family and broader community involvement) shape childhood lifestyles, behaviors, and environmental risk factors associated with CMDs [11, 16, 17]. In a scoping review of community-engaged participatory research with Pacific Islanders in the United States (USA) and the US Affiliated Pacific Islands (USAPIs) between 2000 and 2007, honoring Pacific Islanders’ cultural practices was identified as among the best working practices [12]. When working with Pacific Islander communities, protocols for engagement, reciprocity, and social and spiritual inclusiveness included sharing meals, use of faith-based approaches, and research methods that respected Pacific oral traditions and preference for a “talk story” style of communication [12].

With this in mind, a scoping review is necessary to identify and summarize existing multi-component interventions for Pacific Islanders that could be potentially replicated or adapted for use in Pacific Islander communities in future research. Since our preliminary search of Scopus reported no published or ongoing reviews on this topic, we will review all published original studies, reports, and grey literature that discuss multicomponent interventions to address CMD risk among Pacific Islander children in the USA, the USAPIs, other countries with a large portion of residents who identify as Pacific Islander (e.g. Australia and Aotearoa New Zealand), and three Pacific zones (Micronesia, Melanesia, and Polynesia).

The scoping review questions include, but are not limited to:

1. What are the existing multicomponent intervention designs in published original studies, reports, and grey literature?
2. Were the methods developed within a Pacific-focused research framework and/or theoretical model for implementation science?
3. How were CMD risk factors (including, but not limited to, hyperglycemia/diabetes, high blood pressure/hypertension, dyslipidemia/lipid abnormalities, obesity, and health behaviors) measured and defined in multicomponent interventions for Pacific Islander children?
4. What lifestyle/behavior changes did the multicomponent interventions focus on?
5. What qualitative and quantitative measures of reported efficacy, feasibility, and acceptability were used? Were data disaggregated by subgroups of Pacific Islanders?
6. What were the reported intervention and implementation outcomes?
7. What knowledge gaps remain in the existing literature?

The evidence synthesis from this scoping review will help to identify best practices from existing multicomponent interventions in Pacific communities that could be replicated or adapted to address CMD risk among Pacific Islander children.

## Materials and methods

The protocol is based on the scoping review methods and reporting guidelines provided by the Joanna Briggs Institute Manual for Evidence Synthesis and the Preferred Reporting Items for Scoping Reviews (PRISMA-ScR) [18–20]. The protocol and search strategy have been posted on the Open Science Framework (OSF).

### Eligibility criteria

Studies will be included in the scoping review if they fulfill the following criteria:

#### Study population

Studies will include Pacific Islander children (ranging in ages from 2 years up to 12 years old) living in the USA, the USAPIs, other countries with a large portion of residents who identify as Pacific Islander (e.g. Australia and Aotearoa New Zealand), or countries in three geographical zones in the Pacific: Polynesia, Micronesia, and Melanesia. The list of included countries or regions follows the WHO definition of Pacific Island Countries [21] and previous protocols with Pacific Islander populations [12, 22], which includes American Samoa, Guam, Hawaiʻi, the Commonwealth of the Northern Mariana Islands (CNMI), the Federated States of Micronesia (FSM), the Republic of the Marshall Islands (RMI), Palau, Kiribati, Nauru, Papua New Guinea, the Solomon Islands, Fiji, New Caledonia, Vanuatu, Tonga, Tuvalu, Tokelau, Niue, French Polynesia, New Zealand, Samoa, Tahiti, and the Cook Islands. Moreover, studies including Māori (the indigenous Polynesian people of Aotearoa New Zealand), Native Hawaiians of Hawaiʻi, and Pacific Islanders in Australia, including those from Ni-Vanuatu and the Pitcairn islands, will be selected.

#### Outcomes of interest

CMD risk factors are the primary outcomes of interest and will include combinations of clinical and behavioral measures as defined by the American Heart Association [23] and recent scientific literature [24]—including obesity, insulin resistance, elevated blood pressure, lipid abnormalities, and health behaviors (including diet, physical activity). Eligible intervention studies will (1) attempt to modify one or more CMD risk factors in Pacific Islander children (ranging in ages from 2 years up to 12 years old), and (2) be multi-component in that they include at least two lifestyle/behavioral change strategies to address CMD risk factors. The study design, research framework, definitions of CMD risk factors, and implementation outcomes of existing interventions in those studies will be reviewed and summarized. We will describe the multiple components of the interventions, for example, the use of positive behavior support methods, parent training programs, and environmental modifications to support behavioral/lifestyle change. Intervention outcomes will be classified as ‘short-term” (immediately once the intervention is completed) or “long-term” (after the intervention).

#### Publication date

We will include studies published in the 10 years prior to search completion, because of advances in intervention research and implementation science during this period [1, 2] and to minimize overlap with prior reviews [1, 4, 5]. Each database will be searched in 2022 for records published on or after January 1, 2012.

#### Publication type

To represent the most recent and important work in the field of multicomponent interventions, this scoping review will include original research studies and study protocols published in peer-reviewed journals, government reports, and other grey literature. There are Pacific Journals, for example, Pacific Health Dialog, Papua New Guinea Medical Journal, and Journal of Samoan Studies, that are not yet indexed in PubMed. Locally implemented programs may not be reported in academic journals, but rather in state or government reports. Clinical trial registrations (such as those in the US clinicaltrials.gov or the equivalent in other settings), dissertations, conference abstracts, and masters’ theses will be eligible for inclusion.

#### Language

Studies published in English will be included. If a study is written in a language other than English, but the title and abstracts are in English, we will include the paper in the title abstract screening stage and attempt to obtain a translation of the full text should it be determined to be relevant to our outcomes of interest.

### Search strategy

Literature search strategies will be developed using three concepts: (1) CMD risk factors (e.g., obesity, hyperglycemia/diabetes, high blood pressure/hypertension, dyslipidemia, and/or health behaviors), (2) children, and (3) Pacific Islander populations. This search strategy can retrieve potentially-relevant papers even if the authors did not explicitly frame their work as “multicomponent interventions.” The search strategy was developed by the first author (CCC) in consultation with all co-authors, including a public health librarian (KN). The section of our search that retrieves papers about Pacific Islander populations in the world is based on the search strategy in a previous protocol [22]. The search strategies and histories for all databases will be archived on an OSF project in a reproducible format, including the platform, search terms, date, and number of results. Our search strategy for Scopus is listed in Table 1. While testing iterations of this search strategy, we evaluated the performance of our search terms for multicomponent studies by confirming that multicomponent studies which had been identified by a recently published integrative review of multicomponent approaches to promoting healthy behaviors [25] were indeed retrieved by the relevant query in our search strategy. An independent medical librarian peer-reviewed this search, using the PRESS Guidelines [26].

**Table 1:** Search strategy on Scopus.

Search Terms	Results on 2022-10-05
( ( (TITLE-ABS-KEY (nutrition OR diet OR dietary OR eating OR "self control" OR self-regulat* OR stress OR lifestyle OR "modifiable behavi*" OR "risk behavi*" OR "social support" OR "famil* support" OR "community support" OR "parental support" OR psycho-social OR psychosocial OR "self monitoring" OR "physical activity" OR "physically active" OR "physical inactivity" OR "physically inactive" OR exercise OR "parent* involvement" OR counseling OR counselling OR sedentary OR "screen time" OR sleep OR education) )	1471 results
AND (TITLE-ABS-KEY (obesity OR obese OR overweight OR "body mass index" OR bmi OR weight OR "body composition" OR "waist circumference" OR "upper arm circumference" OR skinfold* OR adiposity OR adipose OR pre-diabetes OR pre-diabetic OR prediabetes OR prediabetic OR diabetes OR diabetic OR "glycated hemoglobin" OR hba1c OR glucose OR "insulin sensitivity" OR hyperglycemi* OR hyperglycaemi* OR "systolic pressure" OR "diastolic pressure" OR "blood pressure" OR hypertensive OR hypertension OR "cardiometabolic risk" OR "cardiovascular risk" OR "cmd risk" OR "cvd risk" OR cardiometabolic OR dyslipidemia* OR dyslipidaemia* OR cholesterol* OR "high density lipoprotein" OR hdl OR "low density lipoprotein" OR ldl OR triglyceride* OR lipid OR lipids OR lipoprotein* OR "metabolic syndrome"))	
AND (TITLE-ABS-KEY (children OR child OR pediatric* OR paediatric* OR school* OR youth* OR girl* OR boy* OR toddler* OR preschool* OR "day care") ) )	
AND (PUBYEAR > 2011) )	
AND ( (TITLE-ABS-KEY (oceania OR australasia OR "oceanic ancestry group" OR pasifika OR pacifica OR melanesia* OR micronesia* OR polynesia* OR hawai* OR "hawai’i" OR "ni’ihau" OR niihau* OR "kaua’i" OR kauai* OR "o’ahu" OR oahu* OR "moloka’i" OR molokai* OR "lana’i" OR lanai* OR "kaho’olawe" OR kahoolawe* OR maui* OR "austral island*" OR "tupua’i island" OR "bass island*" OR australasia* OR australia*-pacific OR "south sea island*" OR "caroline island*" OR carolin* OR carolinian* OR chamorro* OR chuuk* OR "cook island*" OR "easter island*" OR fiji* OR futun* OR guam* OR "i-kiribati" OR kiribati* OR kosrae* OR maori* OR "mariana island*" OR mariana* OR marshall* OR "new caledonia*" OR niue* OR ni-vanuatu OR vanuatu* OR tuvalu* OR tahiti* OR palau* OR nauru* OR "papua new guinea*" OR papua* OR "solomon island*" OR tonga* OR tokelau* OR pitcairn* OR "pitcairn island*" OR pohnpei* OR "phoenix island* " OR "rawaki island*" OR "rapa nui*" OR saipan* OR "american samoa*" OR samoa* OR "new zealand*" OR aotearoa* OR (pacific W/2 island*) OR (pacific W/2 child*) ) ) OR (SRCTITLE (oceania OR australasia OR "oceanic ancestry group" OR pasifika OR pacifica OR melanesia* OR micronesia* OR polynesia* OR hawai* OR "hawai’i" OR "ni’ihau" OR niihau* OR "kaua’i" OR kauai* OR "o’ahu" OR oahu* OR "moloka’i" OR molokai* OR "lana’i" OR lanai* OR "kaho’olawe" OR kahoolawe* OR maui* OR "austral island*" OR "tupua’i island" OR "bass island*" OR australasia* OR australia*-pacific OR "south sea island*" OR "caroline island*" OR carolin* OR carolinian* OR chamorro* OR chuuk* OR "cook island*" OR "easter island*" OR fiji* OR futun* OR guam* OR "i-kiribati" OR kiribati* OR kosrae* OR maori* OR "mariana island*" OR mariana* OR marshall* OR "new caledonia*" OR niue* OR ni-vanuatu OR vanuatu* OR tuvalu* OR tahiti* OR palau* OR nauru* OR "papua new guinea*" OR papua* OR "solomon island*" OR tonga* OR tokelau* OR pitcairn* OR "pitcairn island*" OR pohnpei* OR "phoenix island* " OR "rawaki island*" OR "rapa nui*" OR saipan* OR "american samoa*" OR samoa* OR "new zealand*" OR aotearoa* OR (pacific W/2 island*) OR (pacific W/2 child*) ) ) )	

#### Information sources

We will search the following eight databases: Scopus, MEDLINE ALL (Ovid), EMBASE (Ovid), Web of Science Core Collection as licensed at Yale, Cochrane Library, CINAHL (EBSCOhost), ProQuest Dissertations & Theses Global, and Global Health (EBSCO). Articles published in regional journals across the Pacific will be searched independently.

We will conduct backward and forward citation chaining on papers that meet inclusion criteria and relevant reviews to identify additional studies that may have been missed in the initial search, using CitationChaser to query the bibliographic database Lens [27]. The citation chaining output will be de-duplicated against records that have already been screened as a result of the database searches. Unique records will be screened in Covidence.

We will manually search reports from territorial, state, national, and international agencies, including, but not limited to the Government, Department of Health, Ministry of Health, Pacific Island Health Officers Association, the US Centers for Disease Control, and World Health Organization. This will be completed in the following steps: 1) go to the respective websites of the agencies based on a list developed *a priori* with authors, 2) add to a shared Zotero library documents of potential relevance based on screening questions (that are used for title/abstract screening of bibliographic results in databases), and 3) the full-text of all documents of potential relevance will be screened for eligibility (e.g., at least 2 authors will need to vote ‘yes’ to include the grey literature in the data extraction phase). Similar to prior review studies in other settings [28, 29], we will document the identification and screening process of grey literature to maximize the reproducibility of the information retrieval process. If an author is unable to find any documents of potential relevance on an agency webpage within 30 minutes, then they will document this and proceed to the next agency webpage.

### Data management

We will use Covidence to download and import all search results from databases to deduplicate, title-abstract screen, and full-text screen. We will add other relevant studies identified in hand-searching and citation chaining to the Covidence screening project. We will report the selection process using the PRISMA flowchart.

### Selection of sources and evidence

We will pilot the inclusion and exclusion criteria before the main phase of the title-abstract screening process. For the pilot, at least two authors will review 10–50 records and make title-abstract screening decisions (yes/no). If there are disagreements between the authors, this will be discussed with a senior author to reach a consensus on the interpretation of the inclusion and exclusion criteria.

Screening for each article will occur in two phases: 1) title and abstract screening, and 2) full-text screening. For each phase, at least two authors will review the publications independently. During the screening process, disagreements will be discussed by the first and senior authors to reach a consensus. We record the reason for exclusion during the full-text screening stage. As the review process proceeds, we may record additional exclusion criteria.

Screening questions will include:

1. Was the article or title-abstract published in the English language?
2. Is the article a case report? (Exclude if yes)
3. Does the article discuss an intervention with at least two components addressing lifestyle/behavior change (e.g. counseling on diet, increasing physical activity, or decreasing sedentary behavior)? (Exclude if no)
4. Were children (including ages from 2 years up to 12 years old) the population of interest? (Exclude if no)
5. Were participants included in the intervention identified as Pacific Islander children or children living in the USAPIs, other settings with a large portion of residents who identify as Pacific Islander, or countries in three geographical zones in the Pacific (see eligibility criteria)? (Exclude if no)
6. Were one or more CMD risk factors in children the outcomes of the intervention (e.g. obesity, insulin resistance, dyslipidemia/lipid abnormalities, elevated blood pressure, and/or health behaviors)? (Exclude if no)

### Data extraction process

We will use all articles that meet the eligibility criteria and pass the screening questions. The first author (CCC) will extract relevant data from the articles and compare their data by categories (see Table 2 for examples). The authors will customize a design for a data-extraction sheet that will be used to collect data and relevant information that is consistent with the objectives and research questions of this scoping review. We will pilot the use of this extraction form with several articles to confirm all relevant information is being collected using this process. During the pilot data extraction process, categories will be modified as necessary, and depending on the final included articles, the extraction fields may be subject to change. The senior authors (NH and MS) will review 1 in every 5 extracted manuscripts to ensure the accuracy of data extraction.

**Table 2:** Data extraction categories.

Publication details
• Citation (year of publication and first author)
• Study type (original research article, study protocol, agency report, or grey literature)
• Funding source
Study setting
• Location (country of origin and data source)
• Economic development indicators (e.g., high- vs. middle- vs low-income country)
• Health system characteristics (e.g., number of health care facilities, availability of health care professionals)
• Contextual factors (e.g., existing policies toward interventions of interest, any competing priorities, relationship with academic or community partners)
Intervention characteristics
• Objective(s)
• Type (e.g., community-based or school-based)
• Design and Mode of Delivery (e.g., duration, staff (primary care provider and/or community healthcare workers), 1-on-1 visits, and/or group sessions)
• Components (e.g., Nutrition counseling, use of positive behavior support methods, parent training programs, or physical education)
• Research Frameworks (Pacific-focused vs. not)
• Implementation strategy (e.g., Model of Improvement with Plan-Do-Study-Act cycles or other methods for adaptation)
• Evaluation measures (e.g., intervention acceptability, feasibility, fidelity, reach, cost, the potential for long-term sustainability, and scale-up)
Definition of CMD risk factors
• Measurement methods
• Child growth standards or reference groups
Baseline child characteristics
• Number (Intervention vs. control groups)
• Age/ age range and sex distribution
• Race and ethnicity (e.g., Native Hawaiian, Samoan, or other Pacific Islander subgroups)
• Health status
• Family or household socioeconomic position (education, income, head of household)
Intervention and Implementation outcomes
• Change in body size (e.g., weight or BMI) or CMD risk factors (e.g., glycated hemoglobin, lipid tests, blood pressure, and/or health behaviors)
• Reported feasibility, acceptability, cost, etc.

### Patient and public involvement

The study design and implementation plan do not involve patients and the public. This study will synthesize previously published work, which may report on patients’ and the public’s experiences. Our study does involve public involvement in the reporting and dissemination of the findings in either media or conferences.

### Study status and timeline

Title-abstract and full-text screening are in progress. Citation chaining and searches in grey literature, government reports, and clinical trial registrations (such as those in clinicaltrials.gov or the equivalent in other settings) are in progress. We expect to complete this scoping review by May 2023.

### Presentation of findings

We will provide a narrative summary of the included multicomponent interventions and discuss how our findings in this scoping review answer our research questions. This summary will highlight the advances and gaps in research, as well as existing methodologies in multicomponent behavioral/lifestyle interventions for CMD risk in children. We will organize extracted data by setting (e.g. USA, USAPI, or Pacific Island country), distinct groups (e.g., Native Hawaiian, Samoan, or other Pacific Islander group), intervention type (e.g., community-based or school-based), and quantitative and qualitative measures (e.g., efficacy, feasibility, and acceptability). Supporting figures and tables will be developed to map the evidence and draw implications for future intervention development and adaptation research.

### Ethics and dissemination

Ethical approval is not required, since we are not collecting primary data but rather analyzing published studies, grey literature, and reports. As part of our dissemination plan, we plan to further discuss the feasibility and fit of the identified, existing multicomponent interventions for continued use among Pacific Islanders. The findings will be disseminated through peer-reviewed publications and conferences, as well as in relevant meetings in local and international settings. If any amendments to the protocol are made following its publication, we will provide the date of each amendment, describe the change(s), and report the rationale for the change(s) in future publications arising from this protocol.

## Discussion

This scoping review will synthesize and map existing multicomponent interventions to reduce CMD risk among children, with a particular focus on Pacific Islanders.

### Strengths and limitations of study design

The search strategy of this scoping review is designed to identify and summarize multicomponent interventions implemented over the past decade to address CMD risk during childhood among Pacific Islanders and across the Pacific region; this represents a period in which there has been great advancement in intervention research and implementation science [1, 2]. It will be the first scoping review to focus specifically on the use of multicomponent interventions for Pacific Islander children who are in need of innovative strategies to reduce CMD risk. While the identification and synthesis of data will be limited to published articles, and reports found on certain databases, non-indexed regional journals, clinical trial registries, government reports, other grey literature, and references from citation chaining, we will conform to rigorous methodology manuals of the Joanna Briggs Institute and PRISMA-ScR to enhance the rigor and reproducibility of this work. Importantly, the planned presentation of findings will allow us to consider local strategies in the Pacific region that may be further improved for effectiveness, better understand what is being done in different settings, and whether any interventions are culturally appropriate for Pacific Islander populations.

We anticipate that the scoping review data will guide future research to address gaps in the literature and to help build a stronger evidence basis for the adaptation, implementation, and evaluation of multicomponent interventions for Pacific Islander children. Interventions developed in the Pacific region, using a Pacific and/or implementation science framework will be prioritized for intervention development and adaptation because behavior change strategies may be better aligned with the cultural values of the Pacific community and implementation/evaluation plans may be easier to replicate.
